# The US FDA's proposed rule on laboratory-developed tests: Impacts on clinical laboratory testing

**DOI:** 10.1016/j.plabm.2024.e00407

**Published:** 2024-05-23

**Authors:** Leslie Smith, Lisa A. Carricaburu, Jonathan R. Genzen

**Affiliations:** a ARUP Laboratories, 500 Chipeta Way, Salt Lake City, UT, 84108, USA; b Department of Pathology, University of Utah Health, 15 N. Medical Drive East, Suite 1100, Salt Lake City, UT, 84112, USA

**Keywords:** Laboratory-developed tests, Food and Drug Administration, Clinical Laboratory Improvement Amendments, Clinical laboratory regulations

## Abstract

**Objectives:**

To solicit quantifiable feedback from clinical laboratorians on the U.S. Food and Drug Administration (FDA) proposed rule to regulate laboratory-developed tests (LDTs) as medical devices.

**Design and Methods:**

A ten-item questionnaire was developed and submitted to clinical laboratory customers of ARUP Laboratories, a national nonprofit clinical laboratory of the University of Utah Department of Pathology.

**Results:**

Of 503 clinical laboratory respondents, only 41 (8 %) support the FDA's proposed rule. 67 % of respondents work in laboratories that perform LDTs and were therefore asked additional questions regarding the proposed rule. 84 % of these respondents believe that the proposed rule will negatively impact their laboratories, while only 3 % believe that they have the financial resources to pay for FDA user fees. 61 % of respondents anticipate removing tests from their laboratory menus if the proposed rule is enacted, while an additional 33 % indicated that they do not yet know. Only 11 % of respondents believe that they would pursue FDA submissions for all of their existing LDTs if the final rule is enacted. The vast majority of respondents (>80 %) were either ‘extremely concerned’ or ‘very concerned’ about the impact of the proposed rule on patient access to essential testing, financial and personnel resources to comply, innovation, the FDA's ability to implement the rule, and send-out costs and test prices.

**Conclusions:**

The majority of clinical laboratorians surveyed do not support the FDA's proposed rule on LDTs and report having insufficient resources to comply with the rule if it is enacted.

## Highlights

• The FDA proposed a rule to regulate laboratory-developed tests.
• A survey was conducted to determine clinical laboratory perceptions of this proposal.
• Only a small minority of clinical laboratorians support the FDA's proposed rule.
• The majority of laboratorians believe their laboratories would be negatively impacted.
• Few laboratorians report having the financial resources to comply with the rule.

## 1 Introduction

On October 3, 2023, the U.S. Food and Drug Administration (FDA) released a proposed rule that would regulate laboratory-developed tests (LDTs) as medical devices if enacted [1]. The FDA considers in vitro diagnostics (IVDs) as “tests done on samples such as blood or tissue that have been taken from the human body” [2]. These are traditionally manufactured as reagents and/or kits that are subject to FDA-clearance/approval and are commercially distributed to clinical laboratories for clinical testing purposes. LDTs, however, are developed and performed within clinical laboratories and are not commercially distributed outside of the testing site. The FDA has defined LDTs as IVDs that are “intended for clinical use and designed, manufactured and used within a single laboratory” [3]. While there is no legislative definition from Congress, nor any current federal regulations that create a legal definition of LDTs in the U.S., the FDA's definition has been promulgated extensively in non-binding draft guidance documents and public-facing statements [4,5].

LDTs are not mentioned in the Medical Device Amendments of 1976 [6] – the law that established the current framework for medical device regulation in the U.S. – nor were LDTs discussed in Congressional hearings prior to the law's passage [7,8]. Thus, there is significant legal uncertainty as to whether the FDA has the authority from Congress to advance the current proposed rule [9]. Regardless, FDA leadership has signaled a commitment to move forward with LDT oversight [10], and an April 2024 tentative date for finalization of the proposed rule was posted in the fall 2023 Unified Agenda from the Biden Administration [11,12].

In the U.S., rulemaking from federal agencies must follow requirements outlined in the Administrative Procedure Act [13]. The FDA's proposed rule on LDTs is considered ‘notice-and-comment’ rulemaking, where the public is notified of a proposed rule and provided with the opportunity to provide open comments, and the agency is required to consider this feedback from the public in its deliberation and rulemaking [14]. The public comment period for the FDA's proposed rule was limited to 60 days, and it officially closed on December 4, 2023, with approximately 6700 comments submitted [15]. The FDA declined numerous requests for extensions of the public comment period [16]. These requests included a letter signed by leaders of 89 laboratories and professional organizations [17], as well as a request from the American Medical Association House of Delegates [18]. Extension of public comment periods can provide members of the public more time to evaluate the implication of a proposed rule, and they are often granted by agencies for rules with significant potential impact to the public. The FDA has hosted no public workshops or hearings on LDT oversight since 2015 [19].

An important aspect of the public comment process is to assist a federal agency in understanding whether different sectors of the public support or oppose a proposed rule and for which reasons. While the Federal Register's comment submission system for the proposed rule on LDTs included a drop-down menu for submitters to select the industry, corporate type, career, or setting most applicable to them – presumably to assist federal agencies in categorizing comments by sector – ‘clinical laboratory’ or ‘clinical laboratorian’ were not available options, even though this setting and career type is most impacted by the proposed rule. As such, it will be difficult for the FDA to conduct an accurate quantitative analysis of public opinion in a key sector directly impacted by its proposed rule.

To provide a quantitative assessment of clinical laboratorian opinions on the FDA's proposed rule and its anticipated impact on clinical laboratory operations and patient care, a survey was distributed in February 2024 to customer contacts of ARUP Laboratories. ARUP is a national, nonprofit clinical laboratory enterprise of the University of Utah Department of Pathology, with community hospital and academic medical center laboratory customers across all 50 states [20]. As such, recipients of this survey are directly involved in clinical laboratory testing and have occupational roles for which FDA-cleared/approved IVDs and LDTs are relevant and familiar. Survey results demonstrate that there is strong opposition among clinical laboratory respondents to the proposed rule and significant concerns regarding its anticipated negative impacts on clinical laboratory testing and patient care across laboratories.

## 2 Materials and methods

A ten-item questionnaire regarding the potential impact of the FDA's proposed rule on LDTs was developed (see Survey Questionnaire). The study received an exemption determination (Category 2) from the University of Utah Institutional Review Board (IRB 00174067) [21]. An invitation to participate in this survey was distributed to customer contact email addresses from ARUP's customer relationship management system (CRM) (Salesforce; San Francisco, CA). The survey was opened on January 31, 2024 and closed on February 21, 2024. Documentation of informed consent was waived by the IRB for this exempt protocol. A cover letter outlining the study was provided in the invitation email for this anonymous questionnaire, and individuals who chose to not complete the survey could disregard this invitation. Individual survey links could only be used for one submission. As ARUP Laboratories operates the University of Utah Health clinical laboratories and also serves as the health system's primary reference laboratory, surveys were not distributed to either ARUP or University of Utah Health email addresses to avoid the potential for respondent bias regarding expectations of reference laboratory services. Contacts at national reference laboratories were also excluded to avoid similar bias regarding reference laboratory services. Research trial contacts were also excluded as not all studies require CLIA-certification. The survey was administered using the Experience Management XM platform (Qualtrics; Provo, UT), and no identifiers were collected from survey respondents. Respondent institution type (i.e., community hospital, academic hospital, pediatric hospital, Veterans Administration/federal hospital, independent reference laboratory, pathology group or clinic) and institution location (i.e., U.S. state, territory, or country) was available for a subset of customers in the CRM for summary analysis. A geographic map of institutions in each U.S. state was created in using a template from TheSlideQuest (https://theslidequest.com/) in PowerPoint (Microsoft 365; Redmond, WA).

Data was tabulated within Qualtrics and further analyzed and displayed using Excel (Microsoft 365; Redmond, WA). Percentages are rounded to whole numbers throughout the manuscript. Statistical analyses were performed using R Statistical Software (v4.3.3; R Core Team, 2024). Thematic analysis of free text comments was also conducted and included both semantic themes (e.g., the precise wording of a comment) and latent themes (e.g., an underlying concept in a comment) [22]. Respondent text was assigned to thematic categories and tabulated in Excel, as previously described [23]. Respondent comments displayed in the manuscript were edited for spelling and minor grammatical corrections only.

## 3 Results

**Fig. 1:**
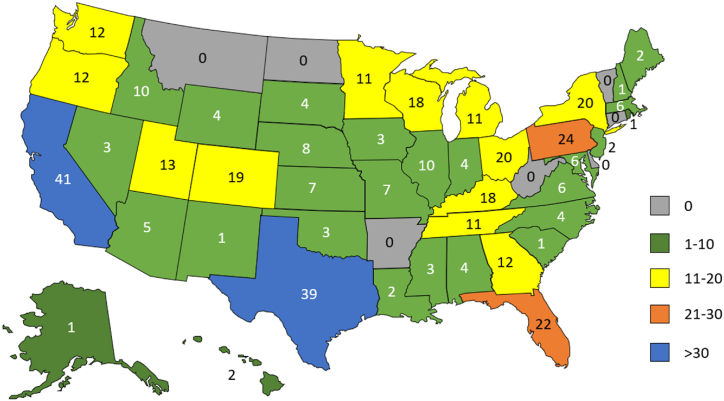
Respondent institution by U.S. state. Map showing respondent institution by state (where available).

Of survey invitations distributed by email to 15,513 individuals, 532 questionnaires (3 % response rate) were either fully or partially completed. Information on respondent institution's state, territory, or country was known for 429 respondents (81 %), with 421 institutions in this subset (98 %) located in the U.S. across 45 states. Eight additional respondents were affiliated with institutions outside the U.S. Distribution of respondent institution location by U.S. state is shown in Fig. 1.

Information on respondent institution type was available for 327 respondents (62 %). Distribution of these institutions by category is shown in Table 1.

**Table 1:** Respondent institution by category.

Institution Category	Number	Percent
Hospital (community)	179	55 %
Hospital (academic)	64	20 %
Independent reference laboratory	40	12 %
Hospital (children's)	25	8 %
Hospital (Veterans Administration/federal)	10	3 %
Pathology group or clinic	9	3 %

Respondents were asked in the questionnaire to specify a job that best describes their roles within their respective organization. A categorized list of respondent job roles is shown in Table 2. The most common job categories selected by respondents included lab manager or supervisor (153, 29 %), lab director (97, 18 %), lab employee (med tech, lab tech, lab assistant; 83, 16 %), and medical director, pathologist, physician, clinician, or PhD scientist (68, 13 %).

**Table 2:** Job categories of survey respondents.

Job Category	Number	Percent
Lab manager or supervisor	153	29 %
Lab director	97	18 %
Lab employee (med tech/lab tech/lab assistant)	83	16 %
Medical director, pathologist, physician, clinician, or PhD scientist	68	13 %
Send-out/referral testing	29	6 %
Quality and compliance	25	5 %
Executive (CEO, CFO, etc.)	23	4 %
IT/LIS	13	2 %
Customer service/support service	10	2 %
Office: executive assistant, administrative assistant, etc.	4	1 %
Specimen processing/receiving	1	0 %
Supply chain/ancillary services	1	0 %
Other	25	5 %
**Total**	532	

Abbreviations: CEO, chief executive officer; CFO, chief financial officer; IT, information technology; LIS, laboratory information system.

**Fig. 2:**
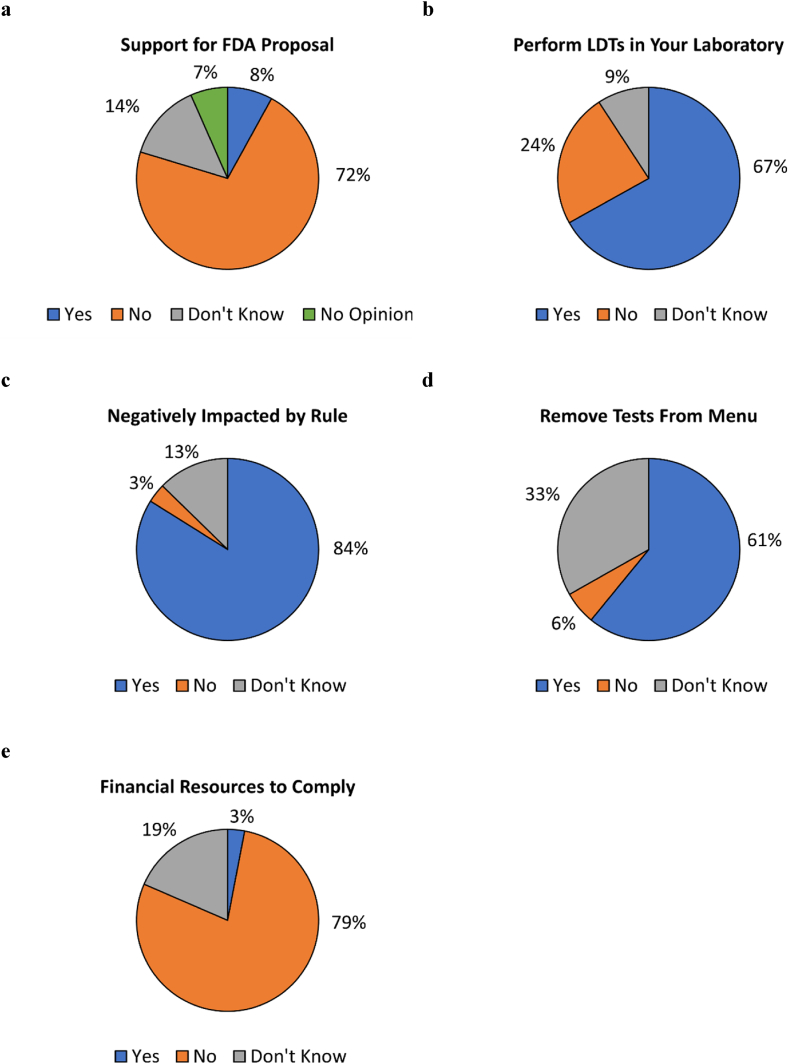
Responses to survey questions. Respondents were asked (a) whether they support the FDA proposal to regulate LDTs, (b) whether they perform LDTs within their laboratories, (c) if their laboratories would be negatively impacted by the proposed rule if enacted, (d) if they anticipate having to remove tests from their laboratory menus if the proposed rule is enacted, and (e) whether they have the financial resources to pay for FDA user fees. Questions for (c), (d), and (e) were asked only of respondents whose laboratories performed LDTs.

503 respondents then answered a question regarding whether they support the FDA's proposed rule to regulate LDTs as medical devices. A majority (360, 72 %) responded ‘no,’ 41 (8 %) responded ‘yes,’ and 102 (20 %) responded that they either had no opinion (69, 14 %) or did not know whether they support the proposed rule (33, 7 %) (Fig. 2a). Distribution of responses according to job category is presented in Table 3. The highest percentage of ‘no’ responses were in the ‘medical director, pathologist, physician, clinician, or PhD scientist’ job category (91 %), the ‘quality and compliance’ job category (91 %), the ‘executive (CEO, CFO, etc.)’ job category (86 %), and the ‘lab director’ job category (83 %). The Fisher's exact test was used to examine the differences in response by job category. Although the test indicated statistical differences within the dataset, pairwise examination of responses by job category found no statistical differences. These conflicting results may be due to a loss of power due to smaller sample sizes in the pairwise analysis.

**Table 3:** Do your support the FDA's proposed rule to regulate laboratory-developed tests and medical devices?

Job Category	Yes		No		Don't Know		No Opinion		Total
	Number	%	Number	%	Number	%	Number	%	Number
Lab manager or supervisor	16	11	101	68	8	5	24	16	149
Lab director	4	4	78	83	4	4	8	9	94
Lab employee (med tech/lab tech/lab assistant)	10	13	45	58	10	13	13	17	78
Medical director, pathologist, physician, clinician, or PhD scientist	0	0	62	91	2	3	4	6	68
Send-out/referral testing	3	12	11	44	4	16	7	28	25
Quality and compliance	1	5	20	91	0	0	1	5	22
Executive (CEO, CFO, etc.)	0	0	19	86	0	0	3	14	22
IT/LIS	2	18	3	27	2	18	4	36	11
Customer service/support service	2	22	6	67	0	0	1	11	9
Office: executive assistant, administrative assistant, etc.	2	67	1	33	0	0	0	0	3
Specimen processing/receiving	0	0	0	0	0	0	1	100	1
Supply chain/ancillary services	1	100	0	0	0	0	0	0	1
Other	0	0	14	70	3	15	3	15	20
**Total**	**41**		**360**		**33**		**69**		**503**

Abbreviations: CEO, chief executive officer; CFO, chief financial officer; IT, information technology; LIS, laboratory information system.

489 respondents then answered a question regarding whether their laboratory performs LDTs. 327 (67 %) responded ‘yes,’ 117 (24 %) responded ‘no,’ and 45 (9 %) responded ‘don't know’ (Fig. 2b). As the survey was intended to assess how laboratories that perform LDTs would respond to the proposed FDA rule if enacted, a response of ‘no’ or ‘don't know’ ended the survey for those respective respondents.

Respondents whose laboratories perform LDTs were then asked whether the FDA's proposed rule would negatively impact their laboratories (n = 322 responses). 270 (84 %) responded ‘yes,’ 11 (3 %) responded ‘no,’ and 41 (13 %) responded ‘don't know’ (Fig. 2c). Respondents were also asked whether they anticipate having to remove tests from their menu if the proposed rule is enacted (n = 304 responses). A majority, 185 (61 %) responded ‘yes,’ 101 (33 %) responded ‘don't know,’ and 18 (6 %) responded ‘no’ (Fig. 2d). Respondents were also asked whether their laboratories have the financial resources to pay for FDA user fees, with fiscal year 2024 medical device user fees displayed in the question stem – $21,760 per “moderate risk” 510(k) submission and $483,560 per “high risk” premarket authorization submission [24] (n = 303 responses). 238 (79 %) responded ‘no,’ 56 (19 %) responded ‘don't know,’ and 9 (3 %) responded ‘yes’ (Fig. 2e).

**Fig. 3:**
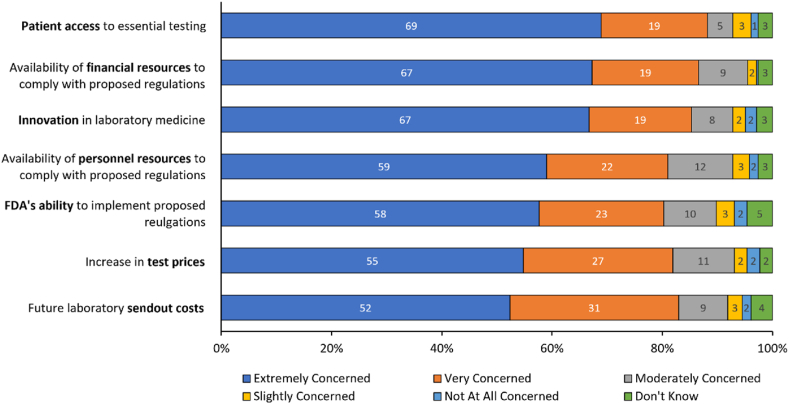
Ratings for level of concern. Stacked bar chart showing respondent level of concern about seven topic areas if the FDA's proposed rule is adopted. Rating scale: extremely concerned, very concerned, moderately concerned, slightly concerned, not at all concerned, don't know. Percentages rounded to zero decimal places.

High levels of concern regarding the potential impact of the proposed rule were expressed by respondents in this survey (Fig. 3), with answers of ‘extremely concerned’ or ‘very concerned’ provided across all topics queried, including patient access to essential testing (269 of 305, 88 %), availability of financial resources to comply with the proposed regulations (264 of 305, 87 %), impact on innovation (261 of 306, 86 %), future laboratory send-out costs (252 of 304, 83 %), increase in test prices (248 of 303, 82 %), availability of personnel resources to comply with the proposed regulations (247 of 305, 81 %), and FDA's ability to implement the proposed regulations (245 of 305, 80 %). Only a small percentage of respondents (≤6 %) chose either ‘not concerned at all’ or ‘slightly concerned’ for any of the topics queried.

**Fig. 4:**
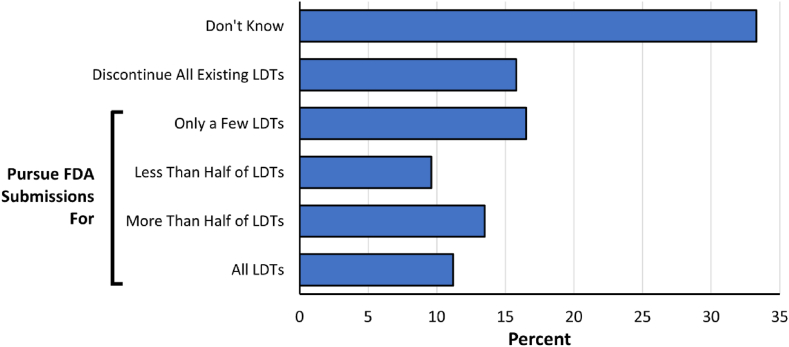
Anticipated response to the proposed rule. Respondents were asked how they think their laboratories would respond to the new regulatory requirements regarding their existing LDTs if the FDA adopts the proposed rule.

When asked how their laboratories would likely respond to the new regulatory requirements if the FDA adopts the proposed rule, respondents reported the following distribution of strategies regarding potential FDA submissions (n = 303 responses): discontinue all existing LDTs that require submissions (48, 16 %); or alternatively pursue FDA submissions for ‘only a few’ of existing LDTs (50, 17 %), ‘less than half’ of existing LDTs (29, 10 %), ‘more than half’ of existing LDTs (41, 14 %), or ‘all’ existing LDTs (34, 11 %) (Fig. 4). One third of respondents (101, 33 %) chose ‘don't know’ to this question, demonstrating that further clarification on the proposed regulations and associated costs will be required to formulate a laboratory's strategy.

**Fig. 5:**
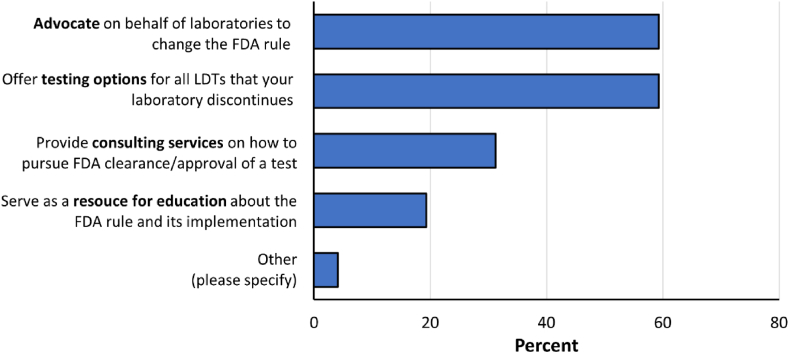
Expected support from reference laboratories. Respondents were asked which types of support they would need from their reference laboratories if the FDA were to adopt the proposed rule. Respondents were instructed to please select their top two types of support.

Respondents were then asked a question regarding expectations of their reference laboratories if the FDA adopts the proposed rule (n = 295 responses; Fig. 5). Respondents were asked to select their top two types of support. An equal majority of respondents expected their reference laboratories to advocate on behalf of laboratories to change the FDA rule (175, 59 %) and to offer testing options for all LDTs that their laboratories discontinue (175, 59.3 %), whereas 92 respondents (31 %) would like consulting services on how to pursue FDA clearance/approval, and 57 respondents (19 %) would like their reference laboratory to serve as a resource for education about the FDA rule and its implementation. 12 respondents (4 %) selected ‘other’ and included a variety of additional ideas including participation in litigation against a final rule, manufacturing and distribution of kits, partnership for specialty testing, sharing of protocols and residual specimens for LDT validation purposes, and physician education to alter LDT ordering practices.

Respondents were then provided an open text comment field to solicit additional feedback. Additional comments were received from 63 respondents. Of these 63 respondents, 1 (2 %) was in support of the FDA's proposed rule, 49 (78 %) expressed opposition to the proposed rule, and 13 (21 %) provided feedback that did not address either support or opposition. Multi-part comments were further subdivided by content to reveal a total of 123 distinct comment topics from respondents for thematic analysis. These were then categorized by semantic and latent thematic categories (Table 4). Representative examples of comments are shown in Table 5.

**Table 4:** **Thematic analysis of open comments.** Thematic categories displayed in the table were mentioned by a minimum of 3 respondents.

Theme	Number	Percent
Negative impact to patients		
- General	18	15
- Pediatric	4	3
Cost & negative economic impact	17	14
Benefits of alternative regulatory structures (e.g., CLIA, CMS, NYSDOH)	12	10
FDA and government overreach	9	7
FDA-specific concerns	8	7
Discontinuation of testing & laboratory closures	6	5
Discipline-specific comments	5	4
Gratitude for survey opportunity	5	4
LDTs are not medical devices/labs are not manufacturers	5	4
Benefits of additional LDT oversight	4	3
Supplier challenges with proposed rule	3	2
Industry disruption	3	2
Other	24	20

Abbreviations: CLIA, Clinical Laboratory Improvement Amendments; LDT, laboratory-developed test; NYSDOH, New York State Department of Health; TAT, turn-around-time.

**Table 5:** Example comments from respondents.

**Patient Care, Cost, and Economic Impact** • If this rule is finalized it is going to adversely impact patient care and health equity. • The rule would even be worse for children's hospitals, as many biochemical tests are performed locally because national reference labs cannot provide the same TATs. • The negative economic impact of the FDA's proposed rule would be immense and has not been accurately or properly represented by the FDA. • Will add significantly to operational costs and lab workforce burnout, thereby creating a significant undue burden, with no data to indicate that this approach will substantively improve safety, efficacy, or utility. • We could not stay in business if we had to pay fees for every test on our menu and then new fees when a test has to be modified because a reagent is no longer available from supplier or in a form it is used in the original procedure.
**Regulatory Comments** • The NYSDOH system for LDT approval is a much better and more reasonable solution to LDT risk assessment. If followed, it would mitigate possible harm from the few LDT's on the market that cause a clinical risk due to poor performance or clinical validity. The FDA proposed rule is akin to a sledgehammer approach to a problem that requires scalpel intervention. • There is a need for oversight of LDTs, but it should be done with updating and modification of CLIA regulations and use that infrastructure. Modernization of CLIA is needed anyway since landscape of all testing (including even point of care) has changed markedly since the 1980s. • LDTs need reasonable regulation, but the FDA is taking a step in the wrong direction. I don't understand why this can't be part of the lab accreditation process, which is already highly scrutinized. Additional burdens to the LDT process make our country less prepared to handle the inevitable next pandemic. • I believe that quality laboratory test validation studies are verified by accrediting organizations (like CAP, COLA, etc.) that have deemed status by CMS. If there is rigor around the inspection process, then FDA oversight of LDTs in an accredited lab is not necessary. • I think having more oversite of LDT's may be warranted, but I am not sure the FDA's approach of requiring approval is reasonable or the most effective way to do this.
**FDA-Specific Comments and Government Overreach** • I am most concerned that the FDA has no genuine interest in listening to the experts (scientists and pathologists) in laboratory medicine. • I don't feel adding FDA oversight would truly improve LDTs, rather it will only increase cost, delay innovation and the speed at which new tests can be put into use which hurts and harms the very persons they are ‘trying to protect’. • I also believe there is no good evidence for the claims or basis of patient harm by LDTs. Again, this appears to be highly misrepresented by the FDA. • The FDA did not fully evaluate the impact this rule would have on labs and to patients. • I am afraid this is another instance of government overreach that does more harm than good. We have CLIA which is currently a way to oversee more closely a laboratory's quality of testing. Adding more layers of bureaucracy and costs does not help anyone. • The FDA is overstepping its mandate and must be curtailed and reined in.
**Discontinuation of Testing, Laboratory Closures, and Impact on Innovation** • The negative impact on our patients will be astounding if we have to divert to send out testing or stop testing all together. • If this regulation were to go into effect, there would be very few tests in our catalog that we could still perform. This would have a significant impact to the community we serve and to public health as a whole. • Due to time and cost constraints, we would likely have to discontinue these tests and stop much of our next generation sequencing tests that serve our complicated patient population which is at a large academic institution. • We truly do not know if we will be able to continue providing local, quality test results to the already under-served patient population. • The financial burdens on small and middle-sized laboratories will be significant and cause many labs to drop important LDTs from their menus. • This will be catastrophic to diagnostic innovation. • This rule could destroy our lab.

Abbreviations: CAP, College of American Pathologists; CLIA, Clinical Laboratory Improvement Amendments; COLA, Commission on Laboratory Accreditation; LDT, laboratory-developed test; NYSDOH, New York State Department of Health; TAT, turn-around-time.

## 4 Discussion

The results of this survey demonstrate that the majority of clinical laboratory respondents are opposed to the FDA's proposed rule on LDTs and believe that their laboratories and patient care will be negatively impacted if the proposed rule is finalized. Most respondents also reported that they do not have the financial resources to pay for FDA user fees, and that they anticipate having to remove existing clinical tests from their laboratory menus if the proposed rule is enacted. This would result in loss of patient access to essential testing services, and/or testing delays due to the send-out of specimens to reference laboratories when testing previously could be conducted in house. Only a minority of respondents reported that they would likely either submit all of their LDTs, or more than half of their LDTs, for review if the FDA adopts the proposed rule. The majority of respondents also expressed strong concern regarding the proposed rule, including its impact on patient access to essential testing, financial and personal resources necessary to comply with the proposed rule, innovation, the FDA's ability to implement the proposed rule, and anticipated increases in send-out costs and test prices.

Statutory language in the Medical Device Amendments of 1976 (related to records and reports on devices) stipulates that the agency “shall not impose requirements unduly burdensome to a device manufacturer, importer, or distributor taking into account his cost of complying with such requirements and the need for the protection of the public health and the implementation of this Act” [25]. As such, the FDA has a legal obligation to consider the cost of proposed regulations in the context of this “unduly burdensome” standard. While the FDA released a regulatory impact analysis in October 2023 to review the purported costs and benefits to society with the proposed rule [26], many organizations (including our own) have noted in public comments that the FDA's analysis includes material errors and assumptions that dramatically under-estimate cost and over-estimate benefit of the proposed rule to society [27,28]. Negative impact to clinical laboratories and patient care was also not thoroughly assessed in the regulatory impact analysis. The present survey provides additional information regarding the perceived burden of the proposed rule on the clinical laboratory community that should be considered by the FDA, the Department of Health and Human Services (HHS), and the White House Office of Information and Regulatory Affairs (OIRA) prior to advancing any final rule, given the potential of the proposed rule for unanticipated negative impacts to clinical laboratory testing and public health [29,30].

Survey results demonstrated that 67 % of respondents perform LDTs within their laboratories. The limited public information on the number and extent of LDTs in use within clinical laboratories has been one justification proposed by the FDA and other groups for additional oversight [26,31]. This does not mean that such information does not exist, however, but rather it is not being collected by federal agencies in a manner that would better inform the public [32]. For example, and as previously discussed [32], all clinical laboratories accredited by the College of American Pathologists (a CLIA-approved accreditation organization) are required to maintain a list of LDTs currently in use [33], although the Centers for Medicare and Medicaid Services (CMS) does not collect this information from the CAP. Additionally, all laboratories accredited by the New York State Department of Health (NYSDOH) are subject to an LDT review and approval process prior to use [34]. NYSDOH also maintains a searchable public database of all approved LDTs offered by its accredited laboratories [35]. Lastly, CMS requires information on assay manufacturers in clinical laboratory CLIA applications [36]. Ultimately, there are already several non-FDA mechanisms for gathering information on how many LDTs are currently in use in the U.S., although CMS has not been active in either collecting or analyzing this information to assist in the current public discourse.

The question of ‘how many’ different types of LDTs are available across clinical laboratories is often confused with the concept of ‘how frequently’ they are ordered by clinicians. In a retrospective study of real-world clinician test orders at a large academic health system, we previously demonstrated that LDTs are ordered far less frequently than is typically portrayed in public discussion [32]. For example, over one year of all test orders in our health system, approximately 94 % of clinician laboratory orders were for FDA-cleared/approved assays, while only 4 % of clinician orders were for LDTs, and 2 % of clinician orders were for standard methods (e.g., differential cell counts, erythrocyte sedimentation rate, urinalysis, Gram stains, etc.) [32]. Many LDTs were associated with test modifications, including alternative specimen types for which the original assay manufacturer did not seek FDA-clearance/approval in its submissions. Under the FDA's proposed rule, the burden for regulatory submissions with common test modifications would fall upon clinical laboratories.

IVD manufacturers typically only distribute FDA-cleared/approved assays that are commercially viable, based on expected order volumes for relatively common disorders and/or use cases. LDTs, however, are often used in situations where FDA-cleared/approved alternatives do not exist, or alternatively when LDTs provide superior analytical performance for a desired use as noted by clinical guidelines. These LDT use cases often represent relatively low-test order volumes in hospital settings, making potential compliance costs with additional regulatory oversight and FDA user fees particularly burdensome. The present survey, for example, demonstrates that only 3 % of clinical laboratory respondents believe that they have the financial resources to comply with existing FDA user fees, and many, if not most, would not qualify for small business discounts as they are incorporated under the hierarchy of much larger health systems. Costs would increase under the proposed rule due to FDA user fees, as well as additional compliance costs to support laboratory quality and technical personnel to conduct revalidations and submit assays in accordance with FDA quality system requirements (QSRs), whereas CLIA QSRs had previously applied to LDTs. A regulatory structure that is cost-prohibitive will unfortunately result in discontinuation of testing options in many settings and potentially closure of some laboratories that are highly dependent upon LDT offerings.

Survey respondents also provided valuable perspectives on what they expect from their reference laboratory partners if the proposed rule is enacted. For example, many respondents (59 %) indicated that they will expect their reference laboratory to offer testing options for all LDTs that their laboratories discontinue. While reference laboratory testing provides an essential service in supporting diagnostic testing needs of providers and patients, transportation of specimens to external laboratories – for testing that could otherwise be routinely performed in-house and closer to the patient – introduces previously avoidable delays in diagnosis and treatment decisions. The negative impact of testing delays to patient care was not, however, factored into the FDA's regulatory impact analysis and deserves careful consideration prior to enactment of any proposed rule.

Many respondents also indicated that they would expect their reference laboratory partner to advocate for changes to the FDA rule on behalf of the respondent laboratory if the proposed rule is adopted. Additional respondents emphasized the desire for education and consultation regarding FDA submissions. Education and advocacy are important elements in reference laboratory partnerships, and this can include a spectrum of activities, including publications [9,[37], [38], [39], [40]], podcasts [41], webinars and online resources [[42], [43], [44]], and public comments and advocacy letters [27,40,45]. An informed clinical laboratory community can better advocate for the needs of its patients, and the survey demonstrates that reference laboratories play an important and ongoing role in these efforts.

Thematic analysis of clinical laboratory respondent free-text responses also revealed significant concerns regarding the proposed rule's impact across multiple domains, including a negative impact to patient care, costs and economic impacts, the benefits of alternative regulatory structures, anticipated discontinuation of testing and laboratory closures, as well as concerns specific to the FDA and an overall perception of government overreach. Numerous comments were directly critical of the FDA and its approach to LDTs, highlighting a considerable lack of trust in the agency by some respondents, at least in the context of the current proposed rule. Potential factors that may contribute to these concerns include clinical laboratorian interactions with the FDA during the COVID-19 pandemic [46,47], unresolved legal questions regarding jurisdiction over LDTs [9], FDA public statements critical of LDTs (and therefore overall quality of testing performed in respondent laboratories) [48], and a disconnect between FDA cost estimates and the financial challenges and staffing constraints within which most clinical laboratories and hospitals operate [49,50]. Deriving a better understanding of the causes for these concerns will be critically important for the success of any future regulatory proposals and should therefore be the subject of further study by the FDA and HHS.

There are several limitations to the present study. The survey distribution included only individuals within one large national reference laboratory's customer contact list, which is a potential source of bias. The results represent the opinions of respondents who agreed to participate in this survey, and there is the potential for selection bias in responses (e.g., individuals who have a vested interest in LDTs may have been more likely to complete the survey). Individuals who may be unfamiliar with LDT regulations or who were busy with other tasks may have been less likely to complete the survey. Additionally, not all laboratory personnel roles may be familiar with the use of LDTs and the expected impacts of potential FDA regulations. As results were anonymized, we could not categorize the job classifications of those who responded ‘other’, nor did we exclude any job classifications to create a more comprehensive overview of clinical laboratory responses. Finally, as multiple extension requests during the public comment period were denied by the FDA, the present results could not be submitted in the public comment submission system, as the portal was closed at the time of study completion. We therefore request that the FDA continue to solicit feedback and work with stakeholders to better understand the negative implications of the proposed rule on clinical laboratories and public health.

## 5 Conclusions

The majority of clinical laboratorians surveyed do not support the FDA's proposed rule on LDTs and report having insufficient resources to comply with the rule if it is enacted. The FDA should work to better understand the negative impacts of the proposed rule prior to finalizing any new oversight structures.

## Funding

This research did not receive any specific grant from funding agencies in the public, commercial, or not-for-profit sectors.

## CRediT authorship contribution statement

**Leslie Smith:** Writing – review & editing, Writing – original draft, Methodology, Formal analysis, Data curation, Conceptualization. **Lisa A. Carricaburu:** Writing – review & editing, Supervision, Resources, Methodology, Investigation, Conceptualization. **Jonathan R. Genzen:** Writing – review & editing, Writing – original draft, Supervision, Project administration, Methodology, Investigation, Formal analysis, Conceptualization.

## Declaration of competing interest

The authors declare the following financial interests/personal relationships which may be considered as potential competing interests:

JRG serves as chief medical officer at ARUP Laboratories and is an employee of the University of Utah; LS is an employee of ARUP Laboratories; LAC is an employee of ARUP Laboratories.

## Data Availability

Data will be made available on request.
