# Supplementary material for: The US FDA's proposed rule on laboratory-developed tests: Impacts on clinical laboratory testing
Source: Pract Lab Med. 2024 May 23;40:e00407. doi: 10.1016/j.plabm.2024.e00407 (PMC11193028; doi:10.1016/j.plabm.2024.e00407)
Supplement: Multimedia component 1 [file mmc1.pdf]

The US FDA’s Proposed Rule on Laboratory-Developed Tests: Impacts on Clinical Laboratory Testing

Survey Questionnaire

Which of the following job categories best describes your role within the organization?

- Customer Service / Support Service
- Executive (CEO, CFO, etc.)
- IT/LIS
- Lab Director
- Lab Employee (Med Tech / Lab Tech / Lab Assistant)
- Lab Manager or Supervisor
- Medical Director, Pathologist, Physician, Clinician, or PhD Scientist
- Office: Executive Assistant, Administrative Assistant, etc.
- Quality and Compliance
- Sendout / Referral Testing
- Specimen Processing / Receiving
- Supply Chain / Ancillary Services
- Other (*please specify*)

Do you support the FDA’s proposed rule to regulate laboratory-developed tests (LDTs) as medical devices?

- Yes
- No
- I don’t have an opinion about this proposed rule
- Don’t know

Does your laboratory perform laboratory-developed tests (LDTs)?

- Yes
- No
- Don’t know

Do you believe your laboratory will be negatively impacted by the FDA’s proposed rule?

- Yes
- No
- Don’t know

The US FDA's Proposed Rule on Laboratory-Developed Tests: Impacts on Clinical Laboratory Testing

Please rate your level of concern about the following if the FDA's proposed rule is adopted: *[Likert Scales; Not at all concerned, Slightly concerned, Moderately concerned, Very concerned, Extremely concerned, Don't know]*

- Patient access to essential testing
- Availability of financial resources to comply with proposed regulations
- Availability of personnel resources to comply with proposed regulations
- Innovation in laboratory medicine
- FDA's ability to implement proposed regulations
- Future laboratory send-out test costs
- Increase in test prices
- Other (*please specify*)

Do you anticipate having to remove tests from your menu if the proposed rule is enacted?

- Yes
- No
- Don't know

If the FDA were to adopt the proposed rule, how do you think your laboratory would likely respond to the new regulatory requirements?

- We would pursue FDA submissions for **all** of our existing LDTs
- We would pursue FDA submissions for **more than half** of our existing LDTs
- We would pursue FDA submissions for **less than half** of our existing LDTs
- We would pursue FDA submissions for **only a few** of our existing LDTs
- We would **discontinue all** of our existing LDTs that require FDA submissions
- Don't know

In your opinion, does your laboratory have the financial resources to pay for FDA user fees? For example, current medical device user fees are $21,760 per "moderate risk" 510(k) submission and $483,560 per "high risk" premarket authorization submission.

- Yes
- No
- Don't know

Which types of support would you need from your reference laboratories if the FDA were to adopt the proposed rule? (*Please select the top 2 types of support*)

- Offer testing options for all LDTs that your laboratory discontinues
- Serve as a resource for education about the FDA rule and its implementation
- Provide consulting services on how to pursue FDA clearance/approval of a test
- Advocate on behalf of laboratories to change the FDA rule
- Other (*please specify*)

Do you have any additional feedback that you did not have an opportunity to share in the survey? [*open text field*]
